# Antibacterial activity and in situ efficacy of *Bidens pilosa* Linn and *Dichrostachys cinerea* Wight et Arn extracts against common diarrhoea-causing waterborne bacteria

**DOI:** 10.1186/s12906-018-2230-9

**Published:** 2018-06-01

**Authors:** Pfarelo Daphney Shandukani, Shonisani Cathphonia Tshidino, Peter Masoko, Kgabo Maureen Moganedi

**Affiliations:** Department of Biochemistry, Microbiology and Biotechnology, University of Limpopo, Private Bag X1106, Sovenga, 0727 South Africa

**Keywords:** Diarrhoea, Simulated gastric fluid, Medicinal plants, Antibacterial activity, Probiotics

## Abstract

**Background:**

*Bidens pilosa* and *Dichrostachys cinerea* extracts were investigated for the antibacterial properties against waterborne diarrhoeagenic bacteria.

**Methods:**

The plant materials were extracted using the direct and serial exhaustive methods using solvents of varying polarities, namely, hexane, dichloromethane, ethyl acetate, acetone and methanol. Qualitative phytochemical analysis and quantitative determination of total phenolic content of the leaf powders of the two plants were tested. The antioxidant activities of the plants were determined using the 2, 2-diphenyl-1-picrylhydrazyl method. The toxic effect of the extracts on C2C12 muscle cell line were assessed by the 3-(4, 5-dimethylthiazol-2-yl)-2, 5-diphenyltetrazolium bromide method and the antibacterial activity was determined using the serial microbroth dilution.

**Results:**

Methanol leaf extracts both plants had the highest yield in both direct and serial exhaustive extraction methods. Phytochemical profiling of the extracts displayed the presence of various secondary metabolites. The Benzene: ethanol: ammonia hydroxide solvent system showed a good resolution of chemical compounds in plant extracts from both plants. Most antioxidant compounds observed were developed in chloroform: ethyl acetate: formic acid and ethyl acetate: methanol: water solvent systems. All the bacterial species tested were sensitive to the effect of different extracts of both plant species, with *E. coli* being less sensitive to the effect of the extracts from *D. cinerea*. Following the simulated gastric fluid (SGF) treatment, a decrease in the antibacterial potency of the extracts was observed. No extract was toxic to the C2C12 muscle cell line.

**Conclusion:**

The presence of the secondary metabolites and nontoxic effect of the two plants tested may affirm the medicinal value of these leaf extracts. Our results suggest that *B. pilosa* and *D. cinerea* contain constituents with antioxidant and antimicrobial activities, which could be used in the treatment of diarrhoea in a case where untreated surface water is used.

## Background

Many communities in South Africa still depend on untreated surface water for their domestic needs such as drinking, cooking, laundry and for personal hygiene [1], due to the shortage of potable water. Surface water is not safe for human consumption and its consumption often results in waterborne diseases such as gastroenteritis, which can be very severe in immunocompromised individuals and children with underdeveloped immune systems [2]. Water-borne diarrhoea and increased bacterial drug-resistance remain a challenge for public health [3]. Diarrhoea constitutes a major cause of morbidity and mortality in South Africa [4], due to widespread usage of non-potable water sources and unhygienic practices. The importance of diarrhoea as a public health problem is marked by relatively reduced number of drugs for its treatment due to their high costs [5]. The high costs of antibiotics [6] and shortage of healthcare facilities in some regions of South Africa [7] leave many affected people with few options for the treatment of infections.

Medicinal plants are commonly used in Africa in the treatment of many ailments and constitute the first health recourse for about 80% of the population [8]. Many rural communities in South Africa still rely on traditional medicines for the treatment of gastric ailments and other diseases [9]. Medicinal plants are rich in compounds that may potentially be natural drugs, which may serve as sources of alternative, affordable and safe antimicrobial treatment for common diseases. Considering the levels of dependency on medicinal plant remedies, scientific research studies have largely reported on the antibacterial and other medicinal properties of the traditional medicinal plants. However, there is less consideration for the investigation of the safety and in situ efficacy of plant extracts used in the treatment of ailments such as diarrhoea. Properties such as potency, possible presence of toxic compounds, spectrum range of the antimicrobial agents and their effect on normal and beneficial flora should be considered with the use of plant preparations. These factors form an integral part in the evaluation process of drug leads. It is thus important to investigate the effectiveness of the plants used for the treatment of diarrhoea, and to further study secondary complications, which may result from the usage of medicinal plants used traditionally to treat diarrhoea based on indigenous knowledge.

*Dichrostachys cinerea* Wight et Arn. and *Bidens pilosa* Linn were investigated in this study due to their wide usage in the treatment of diarrhoea in rural communities. These plant species are used by the vhaVenda people for the treatment of diarrhoea as prescribed by the traditional healers and the herbalists [10], Ramathavha R and Nemakwelengwe M (pers. com). The dried fruits of *D. cinerea* are commonly used as condiments [11, 12], for the management of fever and headache [13] and have been reported to have antioxidant activity [14–16] and anti-hypertensive and antibacterial effects [17, 18].

*Bidens pilosa L.* is a species of flowering plant of the Asteraceae family. In South Africa, *B. pilosa* is found mostly in the wild and homestead gardens in the KwaZulu-Natal and Limpopo Provinces [19]. It is commonly known as Black-jack in South Africa and other countries [20]. Although *B. pilosa* is considered a weed in some parts of the world, in Africa it is commonly consumed as a rich source of food and medicine for human and animals [21, 22]. The aerial parts of *B. pilosa*, i.e., the leaves, flowers, seeds, stems and roots are used in folk medicine as dry powder, decoction, maceration or tincture [23]. The *B. pilosa* extracts have been reported to possess antiviral [24], antifungal [25] and antibacterial properties [26–28].

The purpose of this study was to investigate the antibacterial properties of *D. cinerea* and *B. pilosa* extracts against a wide spectrum of common waterborne diarrhoea-causing bacteria. Notably, *B. pilosa* has not been tested specifically for anti-diarrhoeal efficacy, while *D. cinerea* has been reported [29]. Little information is available on the unintended effects that emanate from consumption of *D. cinerea* preparations*.* It is deemed appropriate that when testing for anti-diarrhoeal properties of medicinal plants, a wide spectrum of appropriate microbiota, which are associated with and are commonly implicated in diarrhoeal episodes in humans are used. It is important to broaden the knowledge on the effectiveness of these selected plants as sources of anti-diarrhoeal agents against common diarrhoea causing bacteria; to support their traditional use and to further shed light on the potential unintended effects associated with consumption of their herbal preparations. The unintended effects of the plant extracts include their effect on probiotics and their retention of antibacterial potency following exposure to gastric fluid.

## Methods

### Plants collection

The selection of medicinal plants was based on their traditional use as reported in Mabogo [10] and from personal communication with herbalists (Ramathavha R and Nemakwelengwe M, pers. com, 2014). Leaves of *B. pilosa* Linn (Voucher specimen no: UNIN 12895) and *D. cinerea* Chiov*,* (Voucher specimen no: UNIN 12894) were collected from Venda (Miluwani village) and Mankweng area (University of Limpopo) in the Limpopo Province. The Larry Leach Herbarium (UNIN) at the University of Limpopo was used as reference point for the identification of plants and as a repository for voucher specimens.

### Plant extraction

Collected plant materials were air-dried in the dark at room temperature for several days, ground with an electric grinder into fine powders and stored in airtight containers. The powdered plant materials were subjected to extraction using the direct and serial exhaustive methods [30, 31]. Briefly, 2 and 5 g of powdered leaves were separately mixed with 20 mL and 50 mL of hexane, dichloromethane, ethyl acetate, acetone and methanol according to the solvents polarities from non-polar to the more polar solvent in polyester centrifuge tubes. The tubes were shaken for 30 min at room temperature in a series 2 incubator shaker (New Brunswick Scientific Co., Inc) at 200 rpm. The plant extract was filtered into pre-weighed labelled vial through a Whatman No. 1 filter paper. The process was repeated three times until the plant constituents were fully collected and the extracts were combined. The solvents were removed under a stream of cold air using a fan. Dried extracts were weighed, recorded and reconstituted in acetone to obtain stock solutions of 100 mg/mL. Extracts were stored as aliquots at − 20 °C until further tests for phytochemicals and antibacterial activity.

### Determination of phytoconstituents

Qualitative phytochemical analyses of *B. pilosa* and *D. cinerea* leaf powders were done for the following classes of secondary metabolites: total phenols [32], terpenoids, steroids and flavonoids [33] and saponins [34].

### Screening for phytochemical compounds

The chemical constituents of the plant extracts were also analysed by thin layer chromatography (TLC) using aluminium-backed TLC plates (Silica gel F254, Fluka) as reported by Kotze and Eloff [30]. Ten microliter of each extract (10 mg/mL) was loaded on TLC plates and developed in saturated chambers using three mobile phases of different polarities; namely, benzene/ethyl acetate/ammonia hydroxide (BEA) (non-polar/basic) (9:1:0.1), chloroform/ethyl acetate/formic acid (CEF) (intermediate polarity/acidic) (5:4:1) and ethyl acetate/methanol/water (EMW) (polar/neutral) (10:5.4:4). The TLC plates were dried in the fume-hood. The developed compounds on the TLC plates were examined under ultraviolet light (254 and 365 nm) and sprayed with vanillin-sulphuric acid reagent [0.1 g vanillin (Sigma ®): 28 mL methanol: 1 mL concentrated sulphuric acid] and heated at 110 °C for optimal colour development.

### Qualitative antioxidant activity

Antioxidant activity was determined using the 2, 2-diphenyl-1-picrylhydrazyl (DPPH) free radical scavenging ability as reported by Deby and Margotteaux [35]. Thin Layer Chromatography (TLC) plates were developed as described above. The plates were sprayed with 0.2% (*w*/*v*) DPPH in methanol as an indicator. The presence of antioxidant compounds was detected by yellow spots against a purple background on the TLC plates.

### Quantitative determination of total phenolic content

The amount of total phenolics in the extracts was quantitatively determined with the Folin-Ciocalteu method [36]. Plant extracts were dissolved in methanol to achieve a concentration of 2 mg/mL. A blank was prepared from all the reagents minus the plant extract. An aliquot of 0.5 mL for each sample was mixed with 2.5 mL of a 10-fold diluted Folin Ciocalteu reagent and 2 mL of 7.5% sodium carbonate in a test tube. The tubes were covered with parafilm and allowed to stand for 30 min at room temperature prior to taking the absorbance reading at 760 nm with the Glomax microtiter plate reader (Promega, U.S.A). Gallic acid was used as a standard at concentrations of 0.01 to 0.05 mg/mL (m/v) in methanol. The total phenolics were expressed as gallic acid equivalent (GAE/mg of extracted material).

### Antibacterial activity of plant extracts

#### Bacterial cultures

The microbiota for the antimicrobial study included the surface water-borne isolate *Klebsiella pneumoniae*, commercial probiotics (combination of *Lactobacillus acidophilus, Lactobacillus casei, Lactobacillus plantarum, Lactobacillus rhamnosus, Lactobacillus salivarius, Lactobacillus bifidum, Lactobacillus breve, Lactobacillus lactis,* and *Lactobacillus thermophillus*), *E. coli* ATCC25922™, *Salmonella typhimurium* ATCC13311™, *Shigella boydii* ATCC9207™ and *Vibrio parahaemolyticus* ATCC17802™. The microorganisms were maintained on Nutrient agar slant at 4 °C and sub-cultured on fresh Nutrient agar plates for 24 h at 37 °C prior to antimicrobial test. The Nutrient broth (NB) was used for the Minimum Inhibition Concentration (MIC) assay.

#### Minimum inhibition concentration

The microplate serial dilution method described by Eloff [31] was used to determine the Minimum Inhibition Concentration (MIC) values of the plant extracts against bacterial pathogens in 96 well microtiter plates. MIC was defined as the lowest concentration of the crude plant extract that inhibits bacterial growth after incubation period. The plant extract was serially diluted with distilled water to a concentration range of 2.5–0.02 mg/mL as described by Eloff [31]. Hundred microliters each of fresh bacterial cultures of probiotics mixture (2.4 × 10^5^ CFU/mL), *S. boydii* (2.4 × 10^5^ CFU/mL) and *S. typhimurium*, *E. coli*, *V. parahaemolyticus* and *K. pneumonia* (1.10 × 10^5^ CFU/mL) were added to the wells of the microtiter plates. Similar serial dilutions were performed for Ampicillin *(*1 mg/mL) as a positive control and acetone as a negative control. Microtiter plates were covered and incubated at 37 °C for 24 h. After incubation, 2 mg/mL of *p-*iodonitrotetrazolium violet (INT) reagent was used as an indicator of bacterial growth.

#### Exposure of plant extracts to simulated gastric fluid

Simulated gastric fluid (SGF) was prepared according to the US Pharmacopeia. Two grams of NaCl, 3.2 g pepsin (Sigma) and 80 mL of 1 M HCl were mixed together to make 1 L with distilled water. Five hundred microliters of each plant extract (10 mg/mL) that had an activity on MIC was incubated with 1 mL of SGF in a shaking incubator at 200 rpm for 2 h at 37 °C. Following incubation, MIC was determined for residual antibacterial activities of the plant extracts.

#### Synergistic activity

The choice of leaf extracts for use in this assay was based on the lowest MIC values obtained in this study for the individual plant extracts. Following the investigation of the independent MIC of the selected plants, the synergistic or antagonistic interactions between the extracts were investigated. Aliquots of 100 μL of bacterial cultures were grown in 100 mL of nutrient broth for 24 h at 37 °C. Different active plant extracts were combined at a ratio of 1:1 (*v*/v). The extract interactions were achieved by determining the MIC of the combinations exhibiting antibacterial activity to establish any interaction effect. The fractional inhibitory concentration (FIC) was calculated for the 1:1 combinations of the plants. This was determined with the equation below, where (i) and (ii) represented the different 1:1 plant combinations [37]. The FIC index was expressed as the sum of FIC _(i)_ and FIC _(ii)_ and this was used to classify the interaction as either synergistic (≤0.50), additive (0.50–1.00), indifferent (> 1.00–4.00) or antagonistic (> 4.00) [38].

$$ FIC(i)\frac{MIC\  of\ (a) in\ combination\ with\ (b)}{MIC\  of\ (a) in dependently} $$

$$ FIC(ii)\frac{MIC\  of\ (b) in\ combination\ with\ (a)}{MIC\  of\ (b) in dependently} $$

#### Evaluation of the cytotoxic activity of the plant extracts on cell culture

The toxic effect of the extracts on muscle cell line C2C12 was assessed by the 3-(4, 5-Dimethylthiazol-2-yl)-2,5-diphenyltetrazolium bromide (MTT) (Sigma®) method [39]. The amount of the insoluble formazan formed from reduction of MTT is directly proportional to the amount of viable cells. Cells were maintained in Dubleco’s Modified Essential Medium (DMEM) supplemented with 10% of Foetal bovine serum (FBS) and 1× Penicillin-Streptomycin-Neomycin (PSN). The C2C12 cells were seeded at a density of 5 × 10^3^ cells/well in a 96 well microtiter plates and incubated overnight at 37 °C in 5% CO_2_. Cells were treated with various concentrations of the extracts with decreasing dilutions from 1000 to 200 μg/mL for 24 h. After 24 h of incubation, the treatment medium was aspirated and 100 μL of MTT at a concentration of 1 mg/mL was added and further incubated for 3 h at 37 °C in the incubator. The formazan product was solubilised in 100 μL of DMSO and was left for 30 min. Spectrophotometric analysis was performed using the Glomax microtiter plate reader (Promega, U.S.A) at 560 nm. Untreated cells with 1000 μg/mL of DMSO served as a negative control and Actinomycin D at a concentration of 40 μg/mL was used as a positive control. The percentage of cell viability was calculated using the [(A/B) × 100] formula, where A is the absorbance value for treated cells and B is the absorbance value for untreated cells.

## Results and discussion

**Fig. 1:**
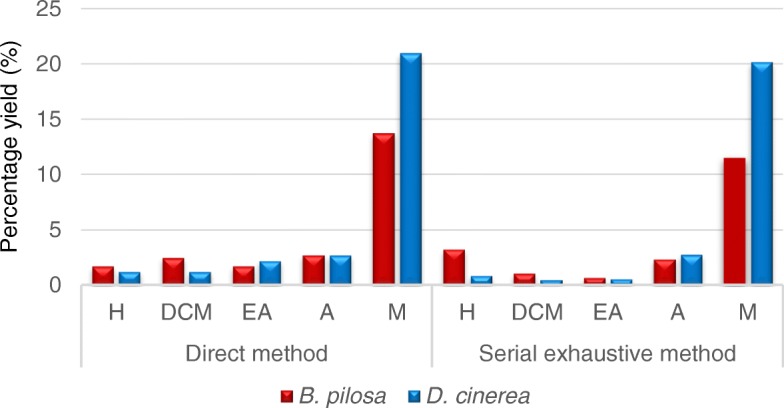
The percentage yield of *B. pilosa* and *D. cinerea* leaf extracts following extraction using the direct and serial exhaustive extraction methods. H, hexane; DCM, dichloromethane; EA, ethyl acetate; A, acetone; M, methanol

**Fig. 2:**
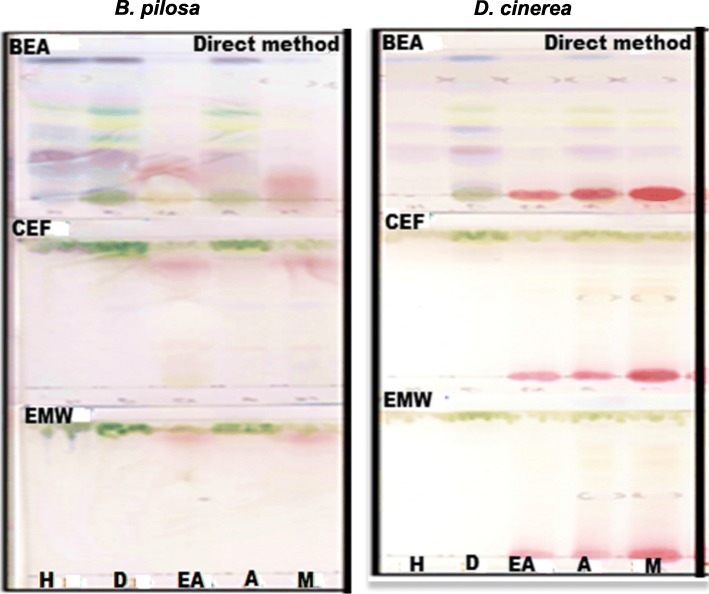
TLC fingerprint profile of plants extracted using the direct extraction method eluted in three solvent systems of varying polarities that show reactive compounds detected by vanillin sulphuric-acid reagent. BEA, Benzene: ethanol: ammonia hydroxide; CEF, Chloroform: ethyl acetate: formic acid; EMW, Ethyl acetate: methanol: water; H, hexane; D, dichloromethane; EA, ethyl acetate; A, acetone; M, methanol

The plant extraction capabilities of the different solvents of varying polarities using the direct and serial exhaustive extraction approaches are indicated in Fig. 1. Extraction is a very important first step in the analysis of medicinal plant properties because the choice of solvent influences the types of compounds that can be extracted and ultimately, the biological activities imparted by the extracted compounds. Two extraction methods were used to check which one will give better yield. After drying the extracts, better yield was obtained when using the direct extraction method than the serial exhaustive method (results not shown). This is in agreement with the study conducted by Masoko et al. [40]. During the phytochemical analysis, more non-polar compounds were reactive to vanillin sulphuric acid reagent than the polar compounds (Fig. 2). This infers that both plant species contain more of non-polar than polar compounds based on the phytochemical profiles.

**Fig. 3:**
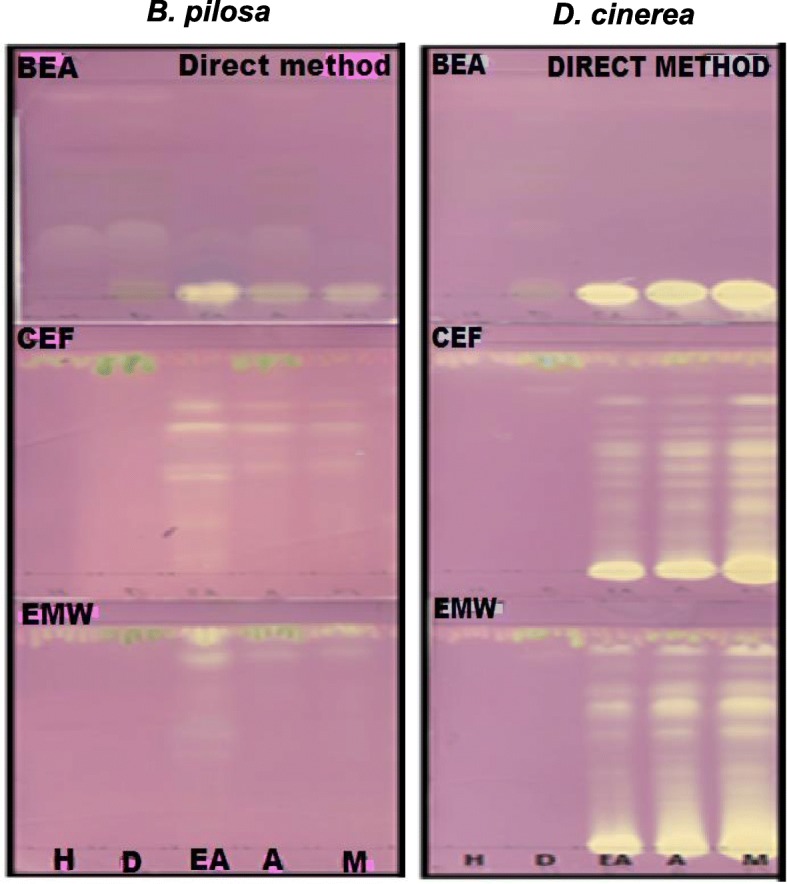
The presence of antioxidant activity depicted by the yellow bands against a purple background. DPPH (0.2%) in methanol was used as an indicator. BEA, Benzene: ethanol: ammonia hydroxide; CEF, Chloroform: ethyl acetate: formic acid; EMW, Ethyl acetate: methanol: water; H, hexane; D, dichloromethane; EA, ethyl acetate; A, acetone; M, methanol

More compounds with antioxidant activity were observed in the intermediate and polar extractants in both plant species, i.e., ethyl acetate, acetone and methanol, which separated well in the polar and intermediate polar mobile systems (Fig. 3). This observation is not uncommon based on the study by Masoko and Eloff [41] and Sudha and Srinivasan [42] who reported the predominant presence of antioxidant compounds in polar than in non-polar plants extracts.

**Fig. 4:**
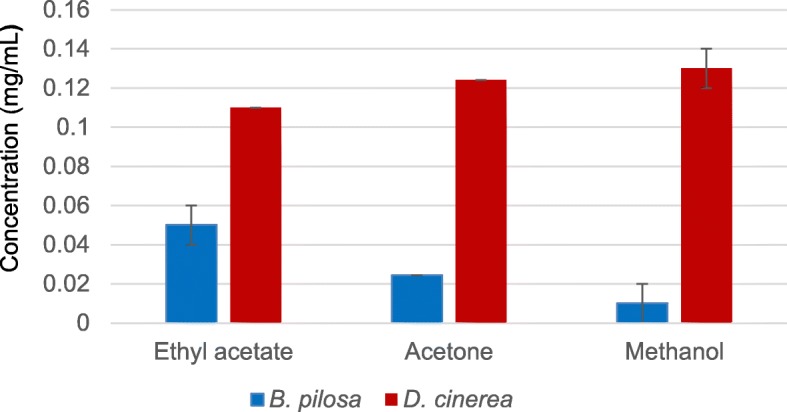
The total phenolic content of leaf extracts of *B. pilosa* and *D. cinerea* extracted by ethyl acetate, acetone and methanol using the direct extraction method

The total phenolic content (Fig. 4) of the leaf extracts of *B. pilosa* and *D. cinerea* was expressed using the gallic acid equivalent. Ethyl acetate, acetone and methanol extracts were selected for determination of the total phenolic content because of the high antioxidant activities of the extracts observed in this study (Fig. 3). Furthermore, phenolic compounds have been reported to exhibit antioxidants activity [43]. The total activity increased with an increase in polarity of the solvent for *D. cinerea* while and the converse was observed for *B. pilosa*. The type of solvent used for extraction plays a role in the ability to extract phenolic components from plant materials [44]. Several studies have shown that methanol and acetone can extract higher amount of phenolics [45–47] and the presence of phenolic compounds might be responsible for the bioactivity of these extracts. The positive association between total phenolic content and antioxidant activity of *B. pilosa* and *D. cinerea* was observed, because the distribution of antioxidant compounds from *D. cinerea* was dominant in the polar to intermediate polar mobile system and the phenolic activity was higher in the polar extracts. On the contrary, the antioxidant activity for *B. pilosa* was apparent in the intermediate to non-polar mobile systems while the total phenolic activity was higher in the intermediate polar extract (Figs. 3 and 4).

The phytochemical compounds assayed in *B. pilosa* showed a predominance of steroids whereas in *D. cinerea* the phenols saponins were the most dominant (Table 1).

**Table 1:** Phytoconstituents of *B. pilosa* and *D. cinerea* leaf extracts

Phytochemicals tested	*B. pilosa*	*D. cinerea*
Phenols	–	+++
Steroids	+++	+
Flavonoids	+	++
Saponins	–	+++
Terpenoids	++	+

- Absent, + slightly present, ++ moderately present, +++ highly present

The antibacterial activity of the medicinal plant extracts varied according to the bacteria tested and the type of solvent used for the extraction. The dichloromethane extracts of both plant species had high antibacterial activity against all the bacterial species tested with an average MIC value of 0.56 mg/mL (Table 2). Nevertheless, *K. pneumoniae* was sensitive to *B. pilosa* and *D. cinerea* extracts but resistant to hexane extracts of both plant species. Ethyl acetate extract of *D. cinerea* showed no activity against most of the tested bacteria, including the probiotics. The dichloromethane extract of both plant species specifically showed higher antibacterial activities against the test organisms, with the most being against the probiotics for *B. pilosa*. The lower antibacterial activities are generally biased towards the non-polar solvents for *B. pilosa*, and this is congruent with the dominance of anti-oxidant activities and phenolic content which separated more in the non-polar solvents as well. It is noteworthy to mention that all the extracts were less potent against the test bacteria due to the observed high MIC values and again the potency of ampicillin which was used as a positive control. Hence, these extracts may not have good candidates as drug leads for antibacterial activity because of these high MIC values due to the importance of the potency of the antibacterial agent in drug development, amongst other factors. Comparatively, the dichloromethane extracts of both plant species gave lower MIC values against all the test organisms and the probiotics, although no antioxidant activity was detected for this solvent in this study. *Shigella* sp*.* was the most susceptible of all the test bacteria with an MIC values of 0.04 mg/mL. The observed antibacterial activity against the probiotics must be considered by the consumers and traditional healers for continuous consumption of the traditional remedies as this may pose a significant health risk in sick patients and can further weaken their immune system. Destabilisation of probiotic activity in the human gut often creates an imbalance of gut flora and this aggravates diarrhoeal episodes. The imbalance of the intestinal flora may consequently lead to the surge of opportunistic pathogens, and this occurrence may inadvertently reverse the beneficial effect of the plant extract, and eventually produce a detrimental effect. Some of the extracts had high MIC values (1.25–2.5 mg/mL) that is an indication of lack of activity against tested microorganisms. The secondary metabolites, namely, terpenoids, steroids and flavonoids were detected in the extracts (Table 1) and these compounds are known to have antibacterial activities [48]. Previous studies has reported antibacterial activity of *D. cinerea* against *Staphylococcus aureus, Bacillus subtilis, Escherichia coli, Pseudomonas aeruginosa* and *Candida albicans* [49]. da Silva et al. [50] also reported antibacterial activity of *B. pilosa* against *Staphylococcus aureus*. The findings in this study that polar solvents such as acetone and methanol produced better antioxidant and antibacterial activities, support the traditional use of water as an extractant for preparations of remedies.

**Table 2:** The MIC values of *B. pilosa* and *D. cinerea* leaf extracts against selected microorganisms

Bacteria	Plants	MIC values (mg/mL)					
		H	D	EA	A	M	Ampicillin
*S. typhimurium*	*B. pilosa*	1.25	1.88	0.31	2.5	2.5	0.23
	*D. cinerea*	2.5	1.25	2.5	1.25	1.25	
*S. boydii*	*B. pilosa*	0.16	0.08	0.94	0.16	0.16	0.04
	*D. cinerea*	1.25	0.04	0.31	0.31	0.16	
*V. parahaemolyticus*	*B. pilosa*	0.31	0.31	0.47	0.47	0.94	0.12
	*D. cinerea*	0.63	1.88	2.5	1.88	2.5	
*E. coli*	*B. pilosa*	1.25	0.08	0.63	0.63	1.25	0.31
	*D. cinerea*	2.5	0.63	2.5	2.5	2.5	
*K. pneumoniae*	*B. pilosa*	2.5	0.12	0.31	0.31	0.31	0.47
	*D. cinerea*	2.5	0.31	0.63	0.16	0.16	
*Probiotics*	*B. pilosa*	0.31	0.01	0.31	0.16	0.16	0.47
	*D. cinerea*	2.5	0.16	2.5	1.25	1.25	
Average		1.47	0.56	1.16	0.97	1.1	

*H* hexane, *D* dichloromethane, *EA* ethyl acetate, *A* acetone, *M* methanol

All the plant extracts that had MIC value ≤1 mg/mL against tested bacteria were exposed to the simulated gastric fluid (SGF). Table 3 shows the results of the plant extracts after exposure to the SGF. *B. pilosa* and *D. cinerea* hexane extracts lost their antibacterial activity following the treatment of SGF. Vermaak et al. [51] and Keating et al. [52] reported that SGF can modify or degrade antibacterial compounds in plants extracts and this can subsequently lead to the loss of initial antibacterial activity in the extracts. In the present study, loss of activity was observed with polar extracts of *D. cinerea* against *S. typhimurium* as compared with the plant extracts alone (Table 3)*.* Interestingly, the gastric fluid played a protective role towards the bacteria by reducing their sensitivity to the extracts. The same effect was observed with ampicillin, which served as the positive control. This protective phenomenon could explain their tenacious survival in the human gut during infections. The results obtained suggest that most of the activities of the orally consumed plant extracts are weakened because of the interaction with the SGF.

**Table 3:** The MIC values of *B. pilosa* and *D. cinerea* leaf extracts after exposure to simulated gastric fluid

Bacteria	Plants	MIC values (mg/mL)					
		H	D	EA	A	M	Ampicillin
*S. typhimurium*	*B. pilosa*	2.5	NA	2.5	NT	NT	0.63
	*D. cinerea*	NA	NA	NT	1.25	1.25	
*S. boydii*	*B. pilosa*	NA	1.25	2.5	1.25	1.25	0.31
	*D. cinerea*	NA	0.63	1.25	0.63	1.25	
*V. parahaemolyticus*	*B. pilosa*	NA	NA	1.25	1.25	1.25	0.31
	*D. cinerea*	NA	NA	NA	2.5	NA	
*E. coli*	*B. pilosa*	NA	1.25	1.25	2.5	NA	0.63
	*D. cinerea*	NT	NA	NT	NT	NA	
*K. pneumoniae*	*B. pilosa*	NT	1.25	2.5	2.5	NA	0.63
	*D. cinerea*	NT	NA	1.25	1.25	1.25	

*NT* Not tested, *NA* No activity, *H* hexane, *D* dichloromethane, *EA* ethyl acetate, *A* acetone, *M* methanol

All extracts with the lowest MIC values for all the bacteria used were further subjected to synergistic analysis. Table 4 shows the results of the MIC values of synergistic activity of *B. pilosa* and *D. cinerea* leaf extracts. Synergy was observed with an FID index of 0.38 for the combination of extracts of *B. pilosa* and *D. cinerea* against *V. parahaemolyticus*, whereas the antibacterial activity of the other combinations did not improve from the independent MIC values of the individual *B. pilosa* and *D. cinerea* extracts.

**Table 4:** MIC values of synergistic activity for *B. pilosa* and *D. cinerea* leaf extracts

Bacteria	*B. pilosa + D. cinerea* (independent MIC, mg/mL)	MIC values in mg/mL (combination)	FIC index
*S. typhimurium*	EA (0.31) + D (1.25)	0.63	2.53
*S. boydii*	D (0.08) + D (0.04)	0.08	3
*V. parahaemolyticus*	H (0.31) + H (0.63)	0.08	0.38
*E. coli*	D (0.08) + D (0.63)	0.16	2.25
*K. pneumoniae*	D (0.12) + D (0.31)	0.31	3.58

*EA* ethyl acetate, *D* dichloromethane, *H* hexane

**Fig. 5:**
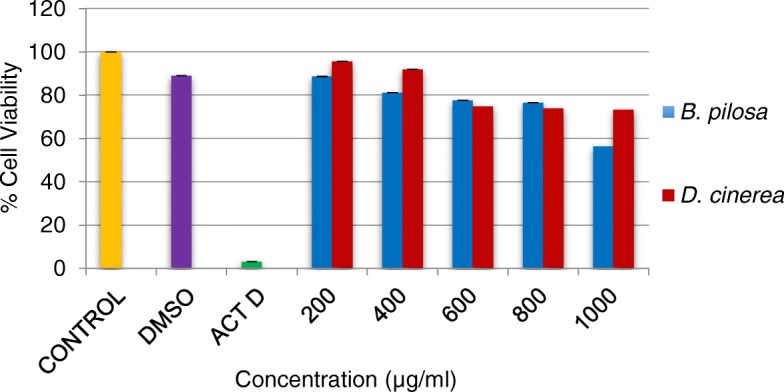
The cytotoxic effects of *B. pilosa* and *D. cinerea* extracts against C2C12

Cytotoxicity effect of the extracts of *D. cinerea* and *B. pilosa* leaves were investigated against C2C12 cell line and the results are shown in Fig. 5. The effects of extracts were compared with the positive control after exposure for 24 and 48 h. None of the crude extracts at different concentrations were toxic to C2C12 cell lines for both plants. Cytotoxicity of these two plants has been investigated for different cell line by other authors and the results showed no effect on the cell line after the treatment [53, 54]. The fact that there was no toxicity observed on the cell line makes the plants extracts beneficial to the rural community of South Africa. This is good especially in the case of *B. pilosa* which is used as a vegetable as part of everyday food by the African people living in the rural villages.

## Conclusion

Waterborne bacterial infections are a serious problem in many developing countries and the decline in fresh water resources aggravates these incidences. Africans preferentially use traditional medicine either as a choice or due to financial constraints wherein western medicine is not an option. While the biological activities of plant extracts can be empirically validated, as was the case in this study, it remains important to investigate other unintended effects that may result in secondary complications from consumption of these medicinal preparations. This current study has shown that leaf extracts of *B. pilosa* and *D. cinerea* are potential sources of antioxidant and antimicrobial agents, which can find application in the treatment of bacterial diarrhoea conditions. Polar solvents such as acetone and methanol showed good antioxidant and antibacterial activities, and this is an advantage to herbalist and traditional healers who use water for preparations of remedies. However, the loss of some antibacterial activity when plant extracts were exposed to gastric fluid is concerning because this may lead to continuous survival of the pathogens in the human gut and prolonged disease episodes. In addition, the adverse effect of plant extracts on the probiotics can be circumvented by re-dosing with probiotics food products such as fermented porridge or other fermented food products which are naturally produced and consumed in many rural African communities.

## Abbreviations

BEA: Benzene: ethanol: ammonia hydroxide
CEF: Chloroform: ethyl acetate: formic acid
DMEM: Dubleco’s Modified Essential Medium
EMW: Ethyl acetate: methanol: water
FBS: Foetal bovine serum
INT: *p-*iodonitrotetrazolium violet
MTT: (4,5-Dimethylthiazol-2-yl)-2,5-diphenyltetrazolium bromide
PSN: Penicillin-Streptomycin-Neomycin
SGF: Simulated gastric fluid
TLC: Thin layer chromatography
